# Outcomes of Endoscopic Closure of Respiratory Esophageal Fistula in Children Using the Esophageal Approach: A Case Series

**DOI:** 10.7759/cureus.29985

**Published:** 2022-10-06

**Authors:** Tawfiq Al Lawati, Omar I Saadah, Mohammed Al Sajwani

**Affiliations:** 1 Pediatrics, The Royal Hospital, Muscat, OMN; 2 Pediatric Gastroenterology, King Abdulaziz University, Jeddah, SAU; 3 Pediatric Surgery, The Royal Hospital, Muscat, OMN

**Keywords:** esophageal pleural fistula, bronchoscopy, endoscopy, esophageal stricture, recurrent tracheoesophageal fistula

## Abstract

Repository-esophageal fistula (REF) in children includes congenital or acquired tracheoesophageal fistula (TEF) and pleuro-esophageal fistula (PEF). TEF is a well-known congenital anomaly that is managed surgically. Recurrent tracheoesophageal fistula (rTEF) occurring after surgical repair of TEF is not an uncommon complication and most of the time requires repeat surgery.

The aim of this paper is to report the outcomes of endoscopic closure of REF in children in Oman. This is a retrospective case series describing the endoscopic closure of REF in children in the Royal Hospital (RH), Oman. Five cases were identified with one of them having acquired PEF while the rest had rTEF. All children had esophageal endoscopic closure of the esophageal fistula using endoclips, cauterization, and glue injection. The patient who had PEF had successful closure of the fistula and only one out of four with rTEF had successful endoscopic closure.

Esophageal endoscopic approach is unsatisfactory in the closure of rTEF but could be effective in the closure of inflammatory PEF. An esophageal approach for the closure of rTEF may need to be consolidated with simultaneous bronchoscopic closure.

## Introduction

Repository-esophageal fistula (REF) in children is mainly congenital as part of congenital esophageal atresia (EA) [1], and less commonly acquired because of infections [2] or foreign body impaction [3]. The incidence of congenital EA, with or without TEF, has been reported to be approximately 1 in 2500-4500 live births [1]. Corrective surgery such as open thoracotomy or thoracoscopic repair is the most commonly used modality for management [4].

Recurrence of esophageal fistula after primary rapier of congenital TEF repair is reported to occur in 3-14% of patients and is likewise managed surgically by open thoracotomy or thoracoscopy, with reported morbidity of up to 60% and mortality of approximately 8% [5,6].

Multiple bronchoscopic techniques have been used to close the TEF, including chemical cauterization, electrocauterization, and fibrin adhesive closure [7,8]. Endoscopic closure using biliary stents applied at the esophagus followed by endoscopic clips was reported in a single infant at three months of age [9]. Another case was reported on a 13-year-old patient that had successful closure of a fistula using histocaryl glue injection at the esophageal end of the fistula after three open surgical attempts [10].

We report here one case of pleuro-esophageal fistula (PEF) and four patients with TEF fistula that had esophageal endoscopic attempts of closure and examine the outcomes.

## Case presentation

Case 1

**Figure 1:**
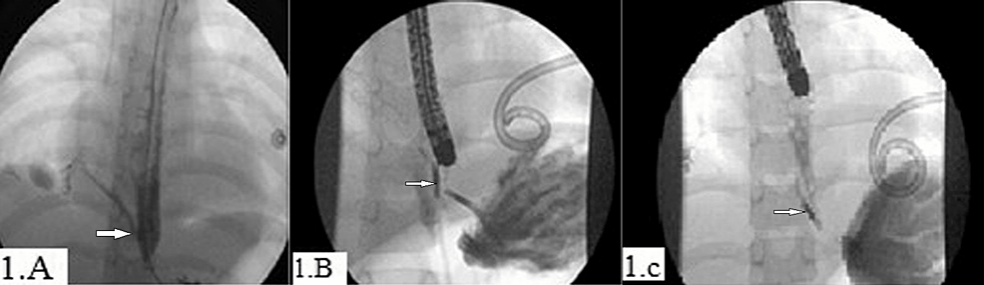
EGD with an intra-operative esophagogram of Case 1 showing inflammatory pleuro-esophageal fistula (A) esophageal pleural leak, (B) application of endoclips, (C) no leak two weeks later EGD: esophagogastroduodenoscopy

M is a 10-year-old male who was referred to the Royal Hospital (RH) with bilateral necrotizing pneumonia, empyema, and multiple pneumatoceles. The pneumonia was further complicated by mediastinitis and inflammatory esophageal perforation causing PEF. M was admitted to the pediatric intensive care unit at the referring hospital and required mechanical ventilation for four weeks. At the RH he continued systemic antibiotics with bilateral insertion of chest drains. Two weeks after admission at the RH, an esophagogastroduodenoscopy (EGD) with an intra-operative esophagogram was carried out that showed a right-sided anterolateral EF draining into the pleural space just above the gastroesophageal junction (Figure 1A). The gastroesophageal junction was at 30 cm from the incisors and the fistula was at 29 cm. Adrenaline (1:20 000, 2 ml) was injected into the fistula site and two endoclips were applied (Figure 1B). No bleeding was noted.

Repeated EGD with intraoperative water-soluble contrast injection in the esophagus two weeks later showed a healed PEF with no contrast leak (Figure 1C). Three days later, the intercostal drains were removed, and the child was discharged on full oral intake.

Case 2

**Figure 2:**
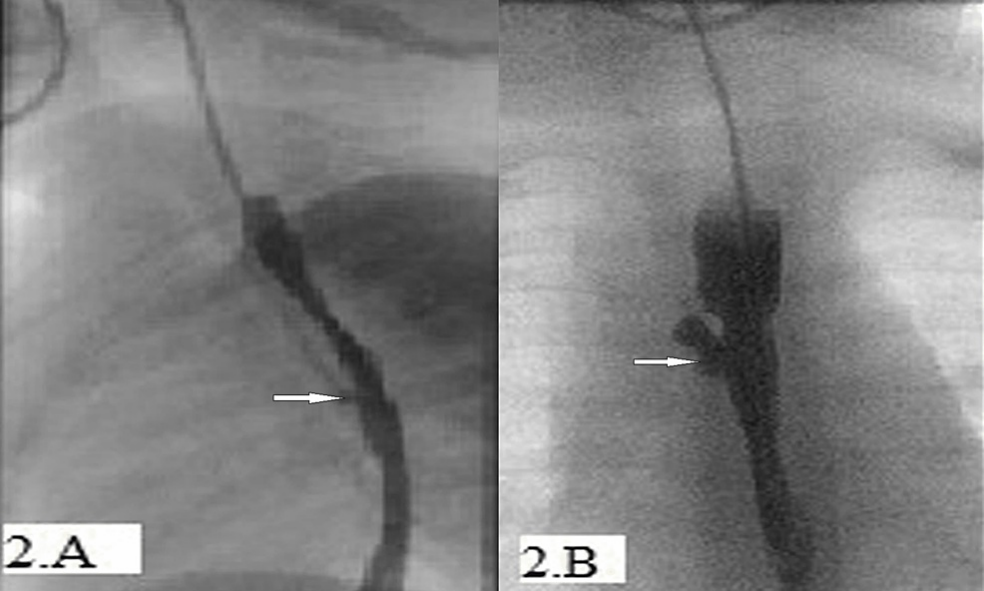
Barium contrast esophagogram study of Case 2 (A) Esophageal contrast leak, (B) esophageal diverticulum with no leak

AS is a one-year-old boy that had type C TEF repair on his second day of life. The procedure was complicated postoperatively with pneumonia, requiring high-frequency ventilation. At the age of four months, the child presented with repeated episodes of cyanosis preceded by a sudden cough during nasogastric tube feeding because of oropharyngeal incoordination. A barium contrast study at the age of six months showed a tight stricture at the site of anastomosis that was dilated using Savory-Gilliard dilators and was complicated by postoperative pneumonia and recurrence of the TEF (Figure 2A).

At the age of seven months, a repeat endoscopy showed the fistula, which was confirmed by an intraoperative esophagogram that also revealed a small diverticulum. Two milliliters (2 ml) of cyanoacrylate polymers were injected at the fistula site using an F23 injector needle. Unfortunately, the fistula persisted post-injection as seen in intraoperative water-soluble contrast injection.

At the age of 10 months, when the child’s weight was around 8 kg, he had a second EGD with argon plasma coagulator (APC) cauterization at the site of the fistula which was followed by glue injection. A repeated barium study two weeks later showed no obliteration of the fistula.

At the age of one year, a third EGD was performed with the application of endoclip at the TEF. The child continued to cough, which was attributed to intermittent aspiration of oral secretions. Two months later a combined EGD and bronchoscopy was carried out using methylene blue injection through the fistula site and showed no tracheal infiltration, confirming closure of the fistula. This was further confirmed by a chest computed tomography scan that revealed no TEF. Barium swallow showed a residual esophageal diverticulum with no leak from the fistula (Figure 2B).

The child continued to have a cough, and a swallow study showed a recurrence of the fistula at the age of two years. Thus, the child underwent surgery for tracheoesophageal repair with no subsequent cough episodes three months following surgery.

Case 3

W is a seven-month-old baby, born at 37 weeks of gestation that had a surgical repair of type C TEF with a 2 cm gap between the two ends of the esophagus on the first day of life. Postoperatively, the child developed sepsis with pseudomonas requiring a prolonged stay in the neonatal intensive care unit.

Barium swallow at the age of one month showed direct oropharyngeal aspiration and recurrence of TEF. An EGD was performed at the age of six weeks which showed a large fistula at the anastomosis site with a tight esophageal stricture. The child was put on nasojejunal feeds, and multiple dilatations were performed as preparation for surgical ligation of the fistula.

At the age of six months, the child had a repeat right thoracotomy for closure of the TEF. Postoperatively, the child again developed pseudomonas pneumonia requiring high-frequency mechanical ventilation.

**Figure 3:**
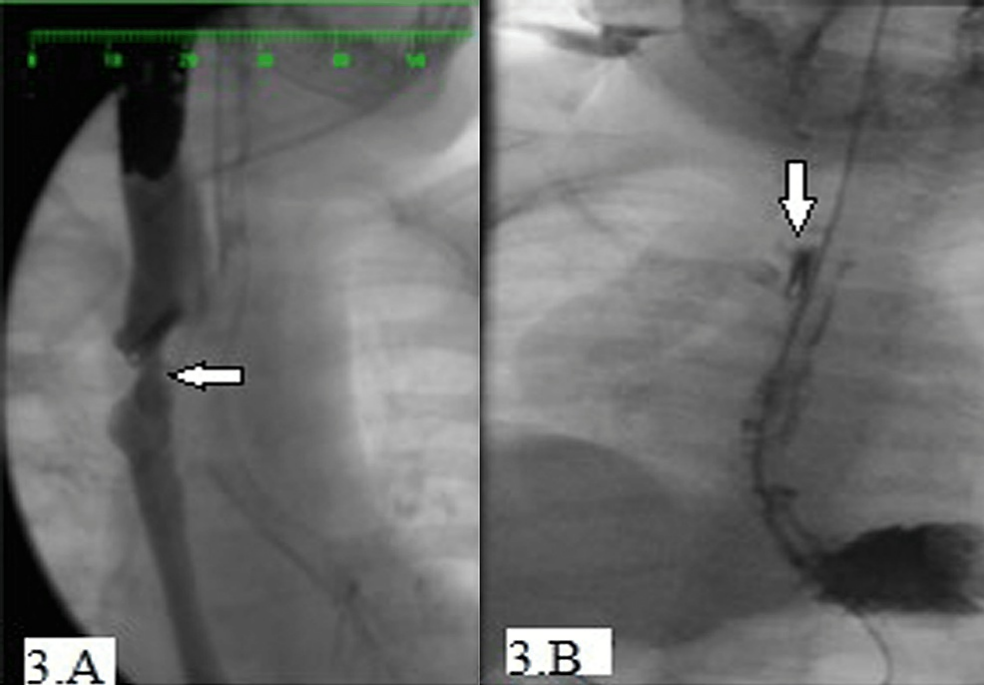
EGD and intraoperative water-soluble contrast of Case 3 (A) Post-operative stricture, (B) post-dilatation esophageal fistula EGD: esophagogastroduodenoscopy

At the age of 10 months, a repeat barium swallow showed a recurrence of TEF. An EGD was carried out and a single adult-sized available endoclip was applied, obliterating the fistula site (Figure 3A). This was followed by a period of three months of protracted nausea and vomiting which was attributed to intolerance to the clips since the TEF was high in the esophagus. A barium swallow two months later confirmed no esophageal leak.

The child continued to have a cough and vomiting and repeated barium swallowing at the age of 16 months showed a recurrence of the fistula (Figure 3B). Eventually, the child underwent surgical repair for the third time.

Case 4

H is a full-term baby from in vitro fertilization, with a birth weight of 3.3 kg that had a TEF type C, with a short gap repaired on the second day of life. The baby remained ventilated for three days and was discharged two weeks later on breastfeeding.

The child was later admitted a number of times for aspiration pneumonia and was noted to have oropharyngeal incoordination. At seven months of age, the baby was found to have a small TEF at the site of surgery using barium swallow, for which he was referred for possible endoscopic management.

**Figure 4:**
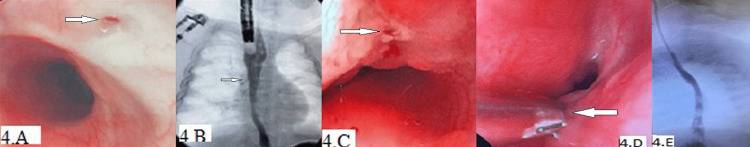
EGDs and Intraoperative water-soluble contrast esophagogram of Case 4 (A) The opening of TEF, (B) contrast leak at the site of TEF, (C) application of APC at the site of the fistula, (D) application of endoclip post cauterization, (E) no contrast leak from the esophagus six weeks later TEF: Tracheoesophageal fistula; EGD: esophagogastroduodenoscopy; APC: argon plasma coagulator

At the age of nine months, the child had his first EGD, revealing a small TEF opening (Figure 4A) with a leak of water-soluble contrast into the trachea (Figure 4B). The fistula was cauterized using an argon plasma coagulator (APC) at the opening and 1.5% of Sotradecol, a sclerosing agent, was injected with elevation and obliteration of the TEF opening (Figure 4C). A repeat esophagogram on the same day showed a persistent leak. A repeat endoscopy six weeks later showed a fistula that was visualized again with a water-soluble contrast injection. The fistula was closed by endoclip application (Figure 4D). The child was discharged post-procedure on nasogastric tube feeds.

Six weeks later, the child had a repeat esophagogram which showed no leak (Figure 4E), and the child was put on oral feeds with no concerns. Ten months later, the child continued to be asymptomatic.

Case 5

**Figure 5:**
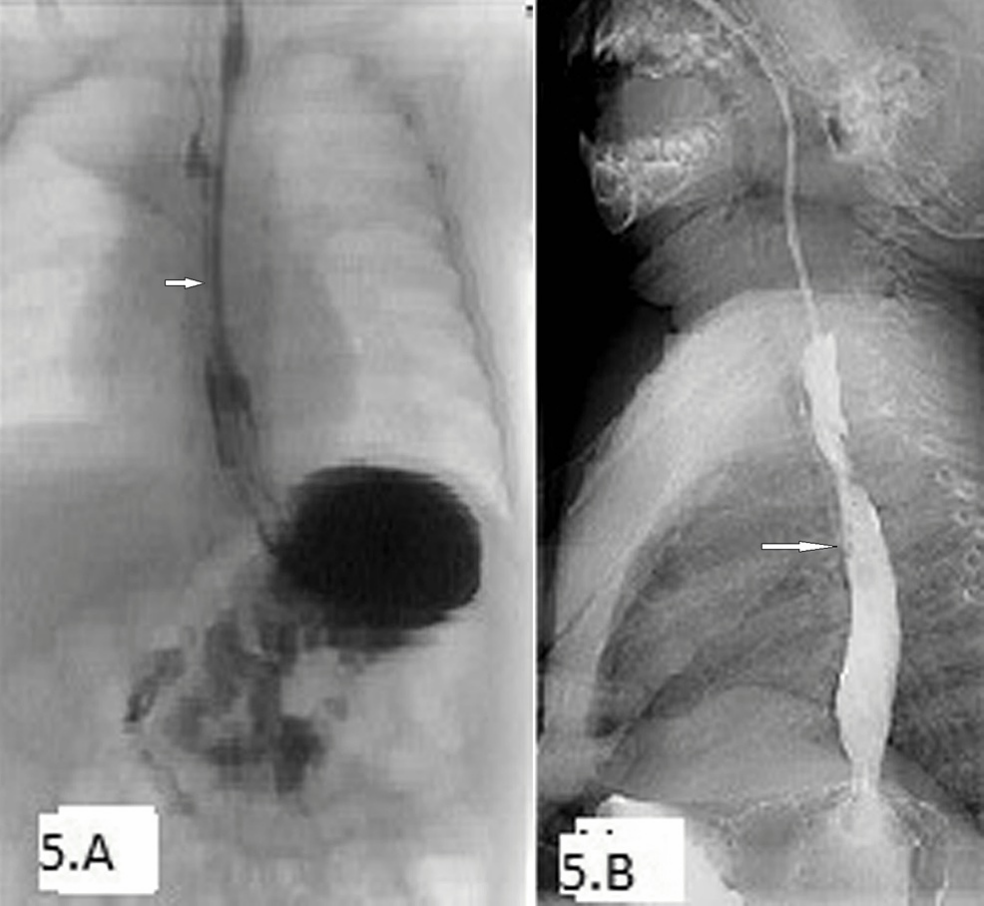
Barium esophagogram of case 5 (A) Esophageal contrast leak, (B) tiny leak on esophagogram

Y is a 10-month-old female baby that was born at term with a birth weight of 2.4 kg with a TEF, a recto-vestibular fistula, and an imperforate anus. She underwent TEF repair on day 2 of life with no post-operative complications. At the age of three months, she presented with pneumonia and was found to have both oropharyngeal incoordination, as well a recurrence of TEF (Figure 5A). Y had an EGD which showed recurrence of the fistula underneath a mucosal flap with a stricture. The TEF was cauterized with an argon plasma coagulation, but the fistula did not heal.

At eight months, Y underwent bronchoscopic cauterization and glue injection at a different hospital with an initial closure of the TEF. At nine months, the child presented again on an esophagogram with a chest infection, demonstrating a recurrence of a tiny TEF (Figure 5B). The child had bronchoscopic cauterization of the fistula, but after one month had a persistent cough with a recurrence of the fistula.

Table 1 illustrates the clinical details of all patients with endoscopic techniques used.

**Table 1:** Clinical details of all the patients and the endoscopic techniques used PEF: pleuro-esophegal fistula; TEF: tracheoesophageal fistula; APC: argon plasma coagulator

Patients	Etiology of fistula	Type of fistula	No. of endoscopic attempts	Techniques used	Age of corrective surgery
Patient 1	Inflammatory	PEF	1	Endoclip and adrenaline injection	Not required
Patient 2	Congenital	Type C TEF	3	Glue application APC cauterization endoclip	2 years
Patient 3	Congenital	Type C TEF	1	Endoclip	16 months
Patient 4	Congenital	Type C TEF	2	Sclerosant cauterization endoclip	2 years
Patient 5	Congenital	Type C TEF	1	Bronchoscopic cauterization and glue application	Planned for surgery at 10 months

## Discussion

This report addressed the outcomes of the endoscopic esophageal approach with respect to EF in five children, finding the successful closure of the fistula in only two cases.

Most of the literature reports on the tracheal rather than the esophageal approach of EF in adults. In a cohort of 25 adults, Silon et. reported the closure of 14 malignant fistulae and 11 benign fistulae using esophageal stenting over the tube clips and endoclips application, with a success rate of 80% [11]. This approach is very difficult in pediatric cases, owing to the unavailability of appropriate endoscopic accessories and the intolerance of children to esophageal stents or clips, particularly in the upper esophagus. In Case 3 of the present study, the child had protracted vomiting and retching because of the high-positioned endoclip. Pierre Goussard reported the use of esophageal stents in three children with TEF with poor outcomes. One child died after putting a stent, the second one had protracted esophageal stricture and the third one had tracheal compression necessitating the removal of the stent [2]. Table 2 lists the studies that used endoscopic closure of TEF in children which are mainly through bronchoscopy [2,12-15]. However, a rare case of an endoscopic esophageal stent using a biliary stent followed by endoclips was reported with success in a three-month-old infant [8].

**Table 2:** Studies report endoscopic management of respiratory esophageal fistulae in children

Authors	No of children	Mean Age	Method	Results	Follow-up period in months
Goussard et al. [2]	8 of 20 cases	34.8 months	Five cases with Brocnoscopic mechanical de-epithelization using cytology brush and fibrin glue; three cases with esophageal stenting	In bronchoscopic closure three out of five achieved closure. In esophageal cases, one mortality after putting a stent, one closed with severe stricture, and one needed stent removal for tracheal compression	6
Valero Mamani et al. [12]	7	4.8 months	Bronchoscopic application of 50% trichloroacetic acid solution	Successful closure	33
Richter et al. [13]	4	11.5 months	Bronchoscopic Bugbee cautery and tissue adhesives	Successful closure	16
Gregory et al. [14]	1	8 months	Bronchoscopic cauterization with Bugbee and Surgisis application in addition to esophageal cauterization	Successful closure	6
Lelonge et al. [15]	12	21.2 months	Bronchoscopic chemical cauterization with 50% trichloroacetic acid	Successful closure of all	46

Unfortunately, we found no studies looking at the esophageal closure of TEF; hence, we could not compare our results with other studies.

Our case series also found that three of the four patients with TEF had significant oropharyngeal incoordination, complicating the issue of aspiration and confusing the management of recurrent aspiration pneumonitis. Oropharyngeal incoordination has been reported in children with recurrent TEF and attributed to esophageal dysmotility [16].

In our experience with EF, the use of injection, cauterization, and endoclip application techniques led to the sustained closure of the fistula in only one child of the four children with TEF and another child with an inflammatory PEF fistula.

We suspect that the oblique position of the TEF, with the tracheal opening being higher (draining the secretin down to the esophagus), the esophageal dysmotility with esophageal strictures, and suboptimal obliteration of the mucosa of the fistula upon application of the APC, all play a role in the failure of fistula closure from the esophageal end.

To overcome the above issues, a tracheal approach via bronchoscopy, brushing off the TEF epithelium vigorously and subsequent application of topical 50% trichloroacetic acid, has shown considerable success [12,15]. It is important also that the bronchoscopic approach can be repeated four to six times before the conclusion of therapy failure may be ascertained. This was not carried out in our patients, often because of parental pressure for definitive treatment.

Acknowledging the high complication rate of anastomotic leakage of 25%, of 61% anastomotic stricture, and of mortality up to 10%, in either open or thoracoscopic surgeries [17], we suggest further studies of combining bronchoscopic and esophageal endoscopic closure approaches with EF. Moreover, these techniques should be further developed by using smaller clips, repeated attempts, and frequent use of brushing techniques.

## Conclusions

Endoscopic management or rTEF using the esophageal approach is not quite effective while it could be effective in inflammatory PEF. Closure from the trachea seems to be more effective in rTEF. New techniques must be devised to improve the outcome of endoscopic management of recurrent TEF to mitigate possible complications of surgery.
